# Mice Lacking Endoglin in Macrophages Show an Impaired Immune Response

**DOI:** 10.1371/journal.pgen.1005935

**Published:** 2016-03-24

**Authors:** Luisa Ojeda-Fernández, Lucía Recio-Poveda, Mikel Aristorena, Pedro Lastres, Francisco J. Blanco, Francisco Sanz-Rodríguez, Eunate Gallardo-Vara, Mateo de las Casas-Engel, Ángel Corbí, Helen M. Arthur, Carmelo Bernabeu, Luisa M. Botella

**Affiliations:** 1 Centro de Investigación en Red de Enfermedades Raras (CIBERER), Valencia, Spain; 2 Centro de Investigaciones Biológicas, Consejo Superior de Investigaciones Biológicas (CSIC), Madrid, Spain; 3 Universidad Autónoma de Madrid, Madrid, Spain; 4 Institute of Genetic Medicine, Newcastle University, Newcastle, United Kingdom; The Jackson Laboratory, UNITED STATES

## Abstract

Endoglin is an auxiliary receptor for members of the TGF-β superfamily and plays an important role in the homeostasis of the vessel wall. Mutations in endoglin gene (*ENG*) or in the closely related TGF-β receptor type I *ACVRL1/ALK1* are responsible for a rare dominant vascular dysplasia, the Hereditary Hemorrhagic Telangiectasia (HHT), or Rendu-Osler-Weber syndrome. Endoglin is also expressed in human macrophages, but its role in macrophage function remains unknown. In this work, we show that endoglin expression is triggered during the monocyte-macrophage differentiation process, both *in vitro* and during the *in vivo* differentiation of blood monocytes recruited to foci of inflammation in wild-type C57BL/6 mice. To analyze the role of endoglin in macrophages *in vivo*, an endoglin myeloid lineage specific knock-out mouse line (*Eng*^*fl/fl*^*LysMCre*) was generated. These mice show a predisposition to develop spontaneous infections by opportunistic bacteria. *Eng*^*fl/fl*^*LysMCre* mice also display increased survival following LPS-induced peritonitis, suggesting a delayed immune response. Phagocytic activity is impaired in peritoneal macrophages, altering one of the main functions of macrophages which contributes to the initiation of the immune response. We also observed altered expression of TGF-β1 target genes in endoglin deficient peritoneal macrophages. Overall, the altered immune activity of endoglin deficient macrophages could help to explain the higher rate of infectious diseases seen in HHT1 patients.

## Author Summary

Endoglin is a transmembrane protein and an auxiliary receptor for TGF-β with an important role in the homeostasis of the vessel wall. However, endoglin was originally identified as a human cell surface antigen expressed in a pre-B leukemic cell line. Mutations in *ENG* are responsible for the Hereditary Hemorrhagic Telangiectasia type 1 (HHT1) or Rendu-Osler-Weber syndrome. HHT is a rare disease, with a prevalence of 1/5,000 to 1/8,000. It is an autosomal dominant disorder characterized by a multisystemic vascular dysplasia, recurrent hemorrhages and arteriovenous malformations in internal organs. Interestingly, endoglin expression is also triggered during the monocyte-macrophage differentiation process. In our laboratory, we described that up-regulation of endoglin during *in vitro* differentiation of blood monocytes is age-dependent and impaired in monocytes from HHT patients, suggesting a role of endoglin in macrophages. In the present work, we first analyzed endoglin expression during differentiation of peripheral blood monocytes to macrophages under *in vitro* and *in vivo* conditions. Next, to investigate endoglin’s role in macrophage function *in vivo*, a myeloid-lineage specific endoglin knock-out mouse line was generated (*Eng*^*fl/fl*^*LysMCre*). Endoglin deficiency in macrophages predisposed animals to spontaneous infections and led to delayed endotoxin-induced mortality. Phagocytic activity by peritoneal macrophages was reduced in the absence of endoglin and altered expression of TGF-β target genes was consistent with an altered balance of TGF-β signaling. The results show a novel role for endoglin in mouse macrophages, which if analogous in human macrophages, may explain, at least in part, the increased infection rates seen in HHT patients.

## Introduction

Endoglin was originally described as a type I integral membrane protein with an extracellular domain of 561 amino acids, a hydrophobic transmembrane domain, and a 47-residue cytosolic domain [1]. It is mainly expressed in endothelial cells and plays a pivotal role modulating cellular responses to TGF-β [1, 2, 3]. Mice lacking endoglin die at E10.5-E11.5 from angiogenic and cardiovascular defects [4, 5, 6]. Mutations in *ENG* are responsible for the Hereditary Hemorrhagic Telangiectasia type 1 (HHT1) [7]. HHT, or Rendu-Osler-Weber syndrome, is a rare disease with a prevalence of 1/5,000 to 1/8,000 and is an autosomal dominant disorder characterized by multisystemic vascular dysplasia and recurrent hemorrhages [8]. In endothelial cells, endoglin promotes a stimulatory effect mediated by TβRII/ALK1 signaling, and inhibitory signals transduced by TβRII/ALK5 signaling complexes [3, 9, 10, 11]. Decreased endoglin expression in endothelial cells from HHT1 donors leads to impaired TGF-β signaling, a disorganized cytoskeleton, and failure to form vascular cord-like structures *in vitro* [12]. In addition, several reports have shown a role for endoglin during hematopoiesis and myeloid lineage development. Endoglin regulates hematopoiesis by modulating the TGF-β signaling pathway in early development [13]. Moreover, in the absence of endoglin, myelopoiesis and definitive erythropoiesis are severely impaired. In contrast, lymphopoiesis appears to be only mildly affected [14]. Furthermore, in endoglin knock-out embryos, hematopoietic colony activity and numbers of erythroid progenitors are severely reduced [15].

TGF-β is a regulatory cytokine with a pivotal role in regulating immune responses [16]. On monocyte/macrophage (Mo/MΦ) cell populations, the action of TGF-β appears to depend on the differentiation stage of the cells. Generally, TGF-β stimulates cells in the resting state (Mo), whereas activated cells (MΦ) are inhibited by it [17]. The role of endoglin on MΦ and immune cell functions remains unknown, although endoglin was identified in differentiated MΦ from human peripheral blood Mo more than 20 years ago [18, 19]. Furthermore, as an extension of this, Sanz-Rodríguez and colleagues [20] described that up-regulation of endoglin during the differentiation of peripheral blood Mo in culture, is age-dependent and impaired in Mo from HHT patients, a fact that could be related to a high frequency of infectious diseases observed in HHT patients [21, 22]. Moreover, endoglin expression in human macrophages is important for blood cell-mediated vascular repair [23]. In addition, colitic *Eng*^*+/-*^ mice show an impaired resolution of inflammation characterized by increased macrophage and neutrophil infiltration, but with a reduction in expression of NAPH oxidase 2 (Nox-2) and myeloperoxidase, two key phagocytic respiratory enzymes [24]. These findings suggest that endoglin is required for fully functional myeloid cells and prompted us to develop a mouse model with endoglin depletion in the myeloid lineage to analyze the role of endoglin in macrophages.

Active TGF-β1 binds to TβRII/ALK5 receptor complex and exerts inhibitory signals for immune cells. The presence of endoglin in MΦ and its capacity to modulate TGF-β signal via ALK1/Smad1/5/8 opens a new door for TGF-β signaling in MΦ. Endoglin expression in human macrophages is well characterized [18,19, 25], but its expression on murine macrophages is not well established. In the present report we describe endoglin up-regulation during *in vitro* transition of murine peripheral blood Mo towards MΦ, and evaluate endoglin expression on tissue-resident MΦ isolated from liver and peritoneal cavity (PerC) of wild-type C57BL/6 mice.

Following the identification of endoglin expression in MΦ, we examined its role in the innate immune system by disrupting endoglin expression in the myeloid lineage *in vivo*. Several HHT mouse models have been developed to investigate the underlying mechanisms leading to vascular malformations in HHT, mainly focusing on the role of endoglin in endothelial cells [26]. Here we present an HHT1 mouse model to dissect the function of endoglin in MΦ, and consequently to elucidate its role in the innate immune response. Mice lacking endoglin expression in MΦ exhibit increased susceptibility to spontaneous infections, an impairment of phagocytic activity, and an aberrant leukocyte recruitment to the site of infection. Endoglin deletion also results in decreased levels of the pro-inflammatory cytokines TNF-α, IL-1β and IL-6, which correlated with a weaker septic response following lipopolysaccharide (LPS) injection. Altogether, these results suggest that endoglin is involved in the regulation of the innate immune response and provide, for the first time, evidence for its role in TGF-β signaling in MΦ *in vivo*. Furthermore, this impairment of the innate immune response seen when endoglin is absent from MΦ may help to explain the high frequency of infectious diseases observed in HHT patients [21, 22].

## Results

### Endoglin is expressed in recently differentiated and tissue resident macrophages

**Fig 1:**
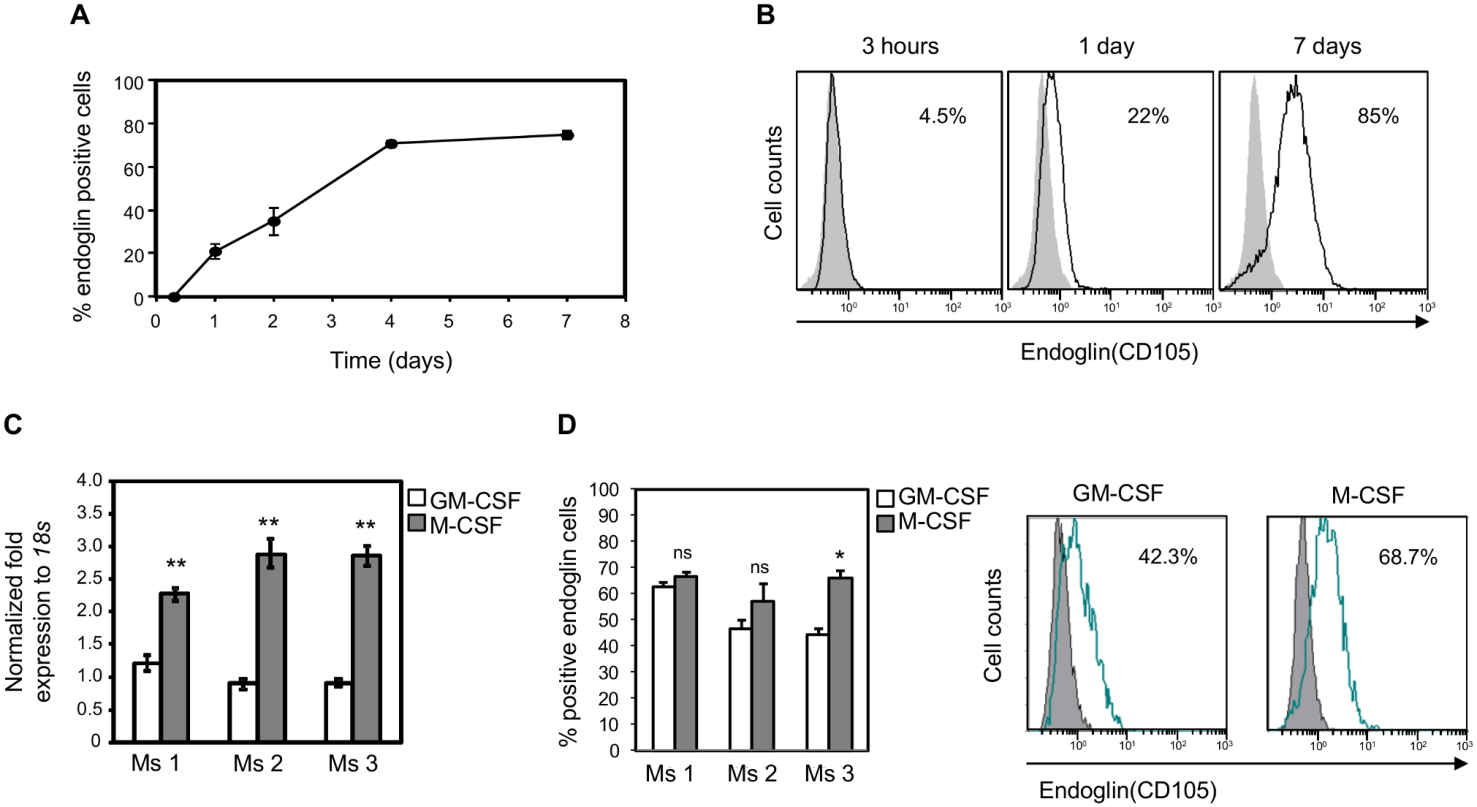
Endoglin expression during *in vitro* differentiation of murine Mo towards MΦ. **(A)** Induction of endoglin expression during *in vitro* cultured blood monocytes. Blood samples from a total of 10 mice for each condition were pooled. Adherent cells were trypsinized at selected times and flow cytometry analysis was used to determine endoglin expression on cultured Mo, defined as CD11b^pos^ Ly6G^neg^ cells. **(B)** Representative flow cytometry graphs of endoglin expression on cultured Mo are shown by profiles with black line. Grey profiles represent the negative control. Percentage of endoglin positive cells is shown in the upper right corner of each histogram **(C)** Endoglin mRNA expression levels in BMDMs differentiated to M1 or M2 macrophages. Expression levels of *Eng* mRNA in BMDMs treated with GM-CSF (M1) or M-CSF (M2) from 3 different C57BL/6 donors. Relative *Eng* mRNA levels are normalized to *18S* as endogenous controls. Mean and SD of each triplicate are shown; ** *P*<0.01 Ms = mouse. **(D)** Endoglin expression levels on BMDMs differentiated to M1 or M2 phenotype from 3 different C57BL/6 mice, with each sample analyzed in duplicate. On the right are representative flow cytometry results showing endoglin expression in BMDMs differentiated to M1 or M2 phenotype. Percentage of positive endoglin cells is shown in the upper right corner of each histogram. Grey profiles represent the negative control.

To follow endoglin expression during *in vitro* differentiation of cultured mouse Mo, flow cytometry analyses of peripheral blood Mo from wild-type mice were carried out. FS vs SS analysis of Peripheral Blood Leukocytes (PBLs) cultured for 3h allowed the identification of Mo as Ly6G^neg^ CD11b^high^ CD3^neg^ population. Three hours after culture, Mo are negative for endoglin expression. Twenty-four hours after culture, 22% of Mo are positive for endoglin expression and there is a gradual increase in the percentage of endoglin positive cells reaching a plateau between 4 and 7 days of 85% (Fig 1A and 1B). In parallel experiments, MΦ were *in vitro* differentiated for 7 days from bone marrow precursors, with GM-CSF or M-CSF to polarize MΦ towards M1 and M2 phenotypes, respectively [27, 28]. Quantitative PCR and flow cytometry analysis showed expression of endoglin in bone marrow derived macrophages (BMDM), both M1 and M2 subtypes from 3 different wild-type mice (Fig 1C and 1D). These data also suggest that endoglin is associated with *in vitro* differentiation of murine MΦ.

**Fig 2:**
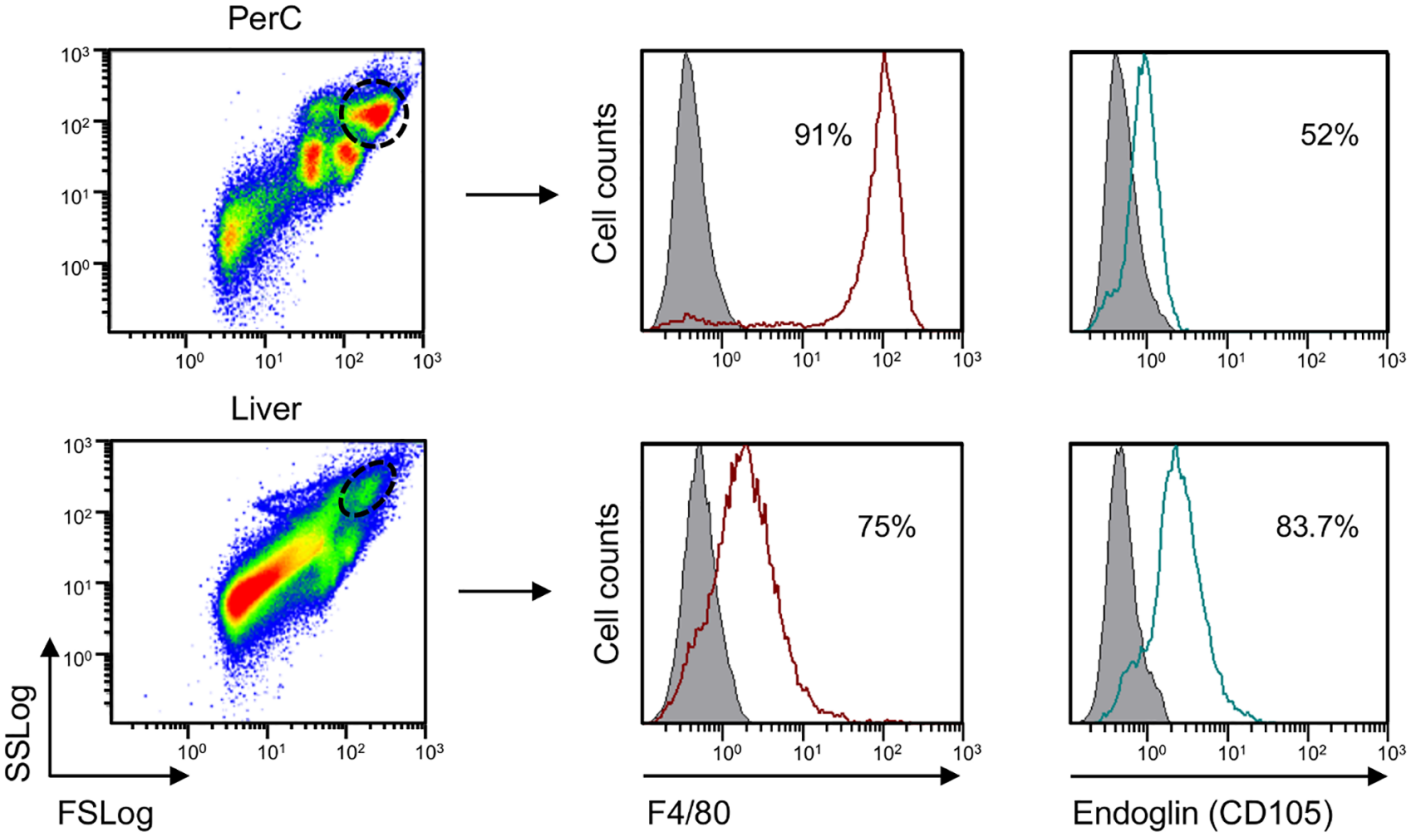
Expression of endoglin in tissue-resident MΦ. Tissue-resident MΦ were isolated from PerC and liver. MΦ were selected by FS vs SS properties. F4/80 and endoglin levels were analyzed on total cells gated in the limited area shown in FS/SS Log plot. The percentage of positive cells for F4/80 and endoglin expression are shown in the upper right corner of each histogram. Negative control is represented by a grey profile. A representative experiment, out of three with similar results, is depicted.

To examine endoglin expression *in vivo*, tissue-resident MΦ were isolated as single cell suspensions from liver and PerC. Cells were analyzed by flow cytometry and endoglin expression was measured on the Ly6G^neg^ F4/80^pos^ cell subpopulation (Fig 2). F4/80, widely used as a mouse MΦ marker [29, 30], is highly expressed on Kupffer cells and resident PerC MΦ, but is weakly expressed in other resident MΦ, as alveolar MΦ or even absent, as in marginal and white pulp splenic MΦ [29, 31]. In the comparative study shown here, F4/80 expression levels are higher in PerC MΦ than in F4/80^pos^ cells present in the liver cellular suspension, likely Kupffer cells. Flow cytometric analysis showed that, PerC MΦ as well as putative Kupffer cells, are positive for endoglin expression. Remarkably, F4/80^pos^ cells from liver express more endoglin than resting PerC macrophages (Fig 2).

### *In vivo* endoglin up-regulation following Mo extravasation to the PerC: ZIP model

**Fig 3:**
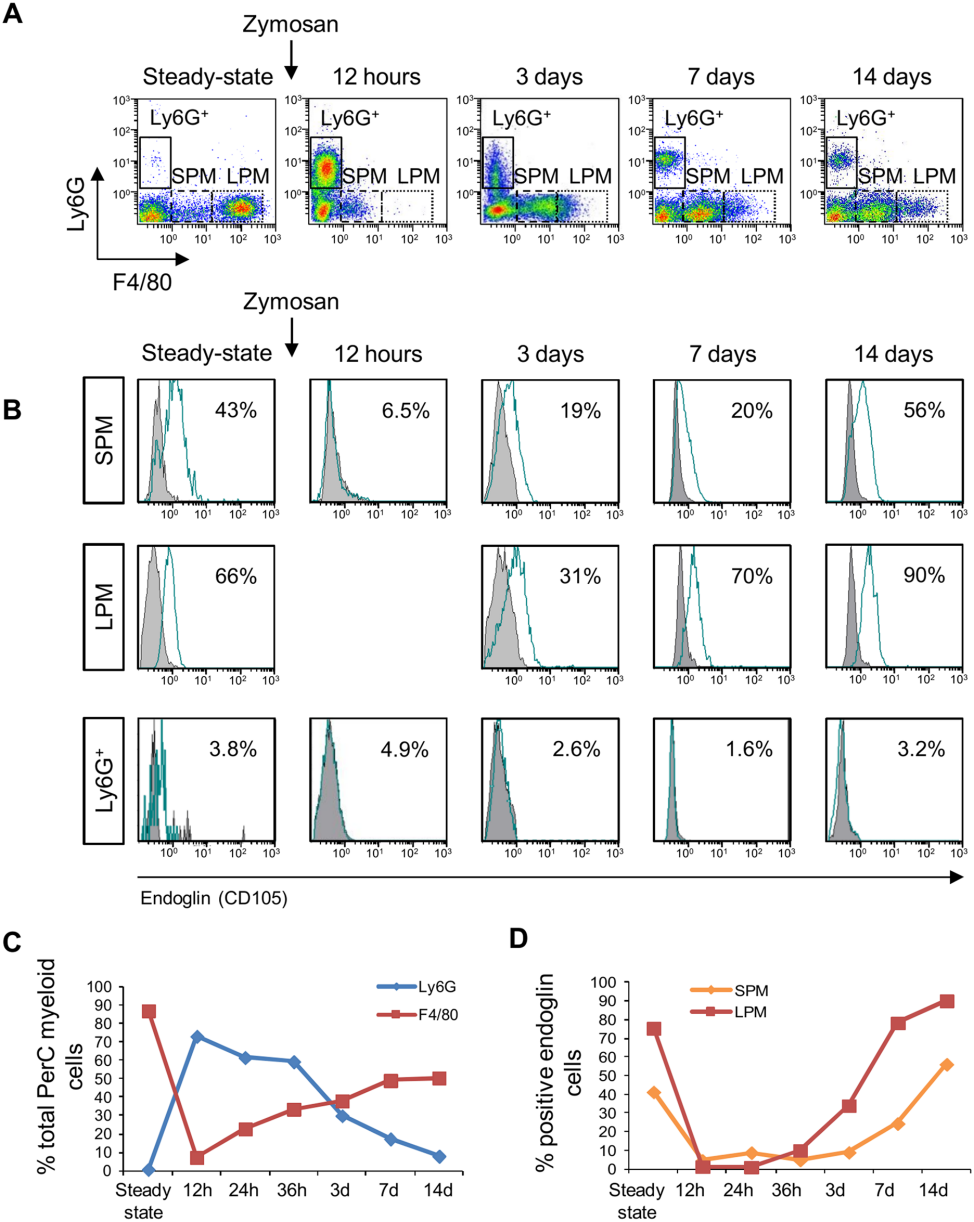
Up-regulation of endoglin expression during *in vivo* Mo recruitment and differentiation to MΦ in the ZIP model. **(A)** Time-course phenotyping of myeloid cells after ZIP. Cells were analyzed by Ly6G vs F4/80 fluorescence intensity. Granulocytes are defined as Ly6G^pos^ F4/80^neg^ (gate shown by full line). MΦ are identified as Ly6G^neg^ F4/80^pos^ and are classified on Small Peritoneal Macrophages (SPM), defined as F4/80^low^ (gate shown by dashed line) and Large Peritoneal Macrophages (LPM), defined as F4/80^high^ (gate shown by dotted lines). Unstimulated mice were used as reference basal conditions (steady-state). PerC cells were isolated after challenge with 1mg of Zymosan A i.p. at selected times (12h, 24h, 36h, 3, 7 or 14 days). **(B)** Endoglin expression in myeloid cells in PerC. Endoglin expression was analyzed on SPM, LPM and granulocytes (defined in panel A) after ZIP. The percentage of positive cells is shown in the upper right corner of each histogram. Negative control is represented by a grey histogram. A pool of 3 animals was analyzed for each condition. A representative experiment, out of three with similar results, is depicted. **(C)** Dynamics of MΦ (SPM+LPM) and granulocytes in PerC during ZIP. **(D)** Time-course of endoglin expression in SPM and LPM during ZIP. The graph shows the percentage of endoglin positive cells in F4/80^low^ (SPM) and F4/80^high^ (LPM). Mean of 2 independent experiments are shown.

Next, we analyzed *in vivo* regulation of endoglin expression in the Mo-MΦ differentiation process. Peritonitis was induced by injecting Zymosan A in the peritoneal cavity, the so called Zymosan induced peritonitis or ZIP model in order to investigate endoglin expression in MΦ derived from peripheral blood Mo and recruited to the PerC. Cells from unstimulated mice (steady-state) were used as controls. Endoglin expression was measured on PerC MΦ defined as Ly6G^neg^ F4/80^pos^, easily distinguishable from the Ly6G^pos^ F4/80^neg^ granulocyte population (Fig 3A). In the PerC, two subpopulations of MΦ have been identified and are defined as Small Peritoneal Macrophages (SPMs) and Large Peritoneal Macrophages (LPMs), due to size differences [31]. They are also characterized by their different F4/80 expression levels: LPMs are Ly6G^neg^ F4/80^high^ while SPMs are Ly6G^neg^ F4/80^low^. In steady-state condition, we observed a main peak of F4/80^high^ expression levels, suggesting that PerC MΦ is highly enriched for LPM, as previously reported for other mouse strains [31]. Until forty-eight hours after 1 mg of Zymosan injection, the predominant PerC MΦ subset is that of SPM, as previously described [32]. As is shown in Fig 3A, twelve hours after ZIP, the main peak of F4/80^pos^ cells corresponds to SPM, and no LPM is detected. Granulocytes do not express endoglin, while endoglin expression in MΦ changes over time (Fig 3B). The first MΦ differentiated from peripheral blood Mo and identified as F4/80^pos^ cells (SPM), are negative for endoglin expression. Indeed, endoglin expression remains undetectable until day 3. At this time point endoglin expression begins to increase after ZIP. Remarkably, there is more endoglin on the brighter F4/80^pos^ cells, likely the LPM macrophage subset, while granulocytes do not express endoglin at all. Thus, endoglin expression is induced during the *in vivo* differentiation process of Mo towards MΦs. These data indicate that the endoglin up-regulation during the Mo to MΦ differentiation process occurs *in vivo*, albeit over a slower time course than observed in *in vitro* assays. The new LPM population is easily distinguishable 2 weeks after ZIP, when the inflammatory response is resolved and the transmigrated Mo are completely differentiated to MΦ and replenish the PerC.

### Generation and characterization of *Eng*^*wt/wt*^*LysMCre*, *Eng*^*wt/fl*^*LysMCre* and *Eng*^*fl/fl*^*LysMCre* mice

**Fig 4:**
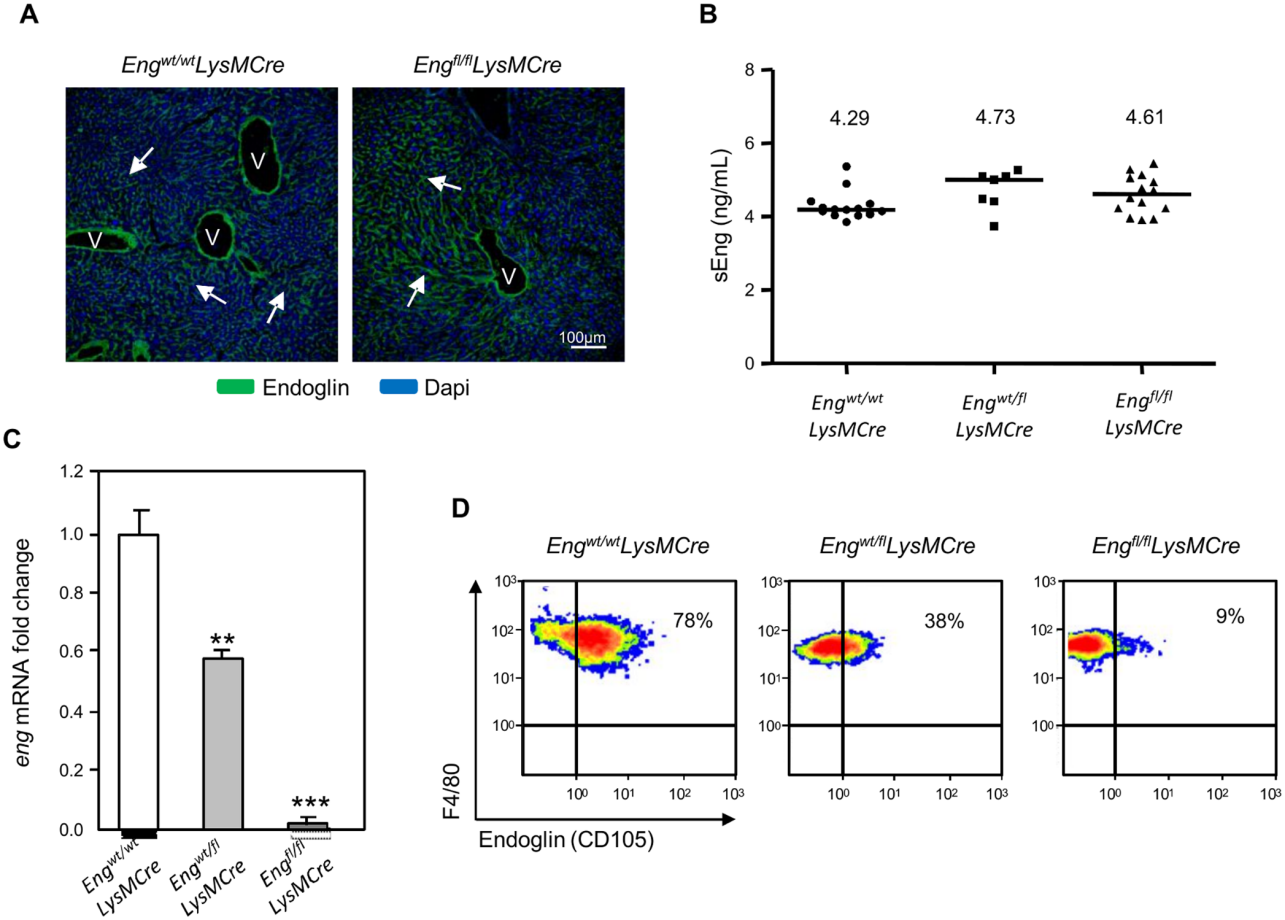
Specificity of LysMCre-mediated lox P recombination in MΦ. **(A)** Confocal microscopy of liver sections from C56BL/6 mice. Endoglin (green) is highly expressed on hepatic sinusoids (arrows) and on endothelium from hepatic vein (V) in *Eng*^*wt/wt*^*LysMCre* and *Eng*^*fl/fl*^*LysMCre*. **(B)** sEng levels in serum samples. Levels of sEng in serum from *Eng*^*wt/wt*^*LysMCre* (n = 14), *Eng*^*wt/fl*^*LysMCre* (n = 7) and *Eng*^*fl/fl*^*LysMCre* mice (n = 14) were determined by ELISA. The mean value of sEng in each group is shown in the upper part. Horizontal line represents the median. ANOVA with Bonferroni’s post-test was performed and no statistical differences were observed. **(C)** qRT-PCR for *Eng* mRNA expression in PerC MΦ. *18s* was used as a reference gene, and data were normalized to *Eng*^wt/wt^*LysMCre* mice. Data are presented as the mean ± SEM and are pooled from five independent experiments (N = 5) with each performed in triplicate; Data were analyzed versus respective controls using Student’s *t* test. **P<0.01 and ***P<0.001. **(D)** Endoglin expression levels in resident PerC MΦ from experimental animals. Flow cytometry plots depict the percentage of endoglin-positive cells among total peritoneal resident MΦ (F4/80^pos^, mainly LPM) in *Eng*^*wt/wt*^*LysMCre*, *Eng*^*wt/fl*^*LysMCre* and *Eng*^*fl/fl*^*LysMCre* mice.

In order to investigate the role of endoglin in MΦ function either in resident tissue MΦ or in activated Mo/MΦ *in* vivo, endoglin specific myeloid lineage knock-out mice were obtained by crossing a strain expressing Cre recombinase from the lysozyme 2 gene (*Lyz2*), the *LysMCre* strain, with a floxed endoglin strain containing *loxP* sites flanking exons 5 & 6, the *Eng*^*fl/fl*^ strain. The scheme of crosses is shown in S1 Fig. The mice were all of the same genetic background (C57BL/6) and were genotyped by genomic PCR using DNA isolated from tail tissue (S1B and S1C Fig). Activity of Cre recombinase was confirmed in MΦ from liver, lung, spleen, heart and peritoneal cavity by the detection of the *Eng*^Δ*5–6*^ allele by genomic PCR in *Eng*^*wt/fl*^*LysMCre* and *Eng*^*fl/fl*^*LysMCre* mice. The specificity of Cre recombinase under the control of the *Lyz2* promoter was checked by immunohistochemistry in liver. The *Lyz2* promoter is specific to the myeloid lineage [30], so it is expected that endoglin expression will be maintained in other cellular types e.g. endothelial cells. In fact, endoglin expression appears unaltered in hepatic sinusoids and in endothelium of hepatic veins of *Eng*^*fl/fl*^*LysMCre* mice (Fig 4A). Moreover, the serum levels of soluble endoglin (sEng), directly related to the levels of endothelial endoglin expression [33], remain unaltered (Fig 4B). The efficiency of *Eng* deletion was complete as confirmed by real-time qPCR analysis of peritoneal MΦ. The *Eng* mRNA levels were almost undetectable in MΦ from *Eng*^*fl/fl*^*LysMCre* mice and were intermediate in *Eng*^*wt/fl*^*LysMCre* mice compared to control mice (Fig 4C). These results were also confirmed at protein level by flow cytometry (Fig 4D).

### *Eng*^*fl/fl*^*LysMCre* mice develop spontaneous infections in soft tissues

**Fig 5:**
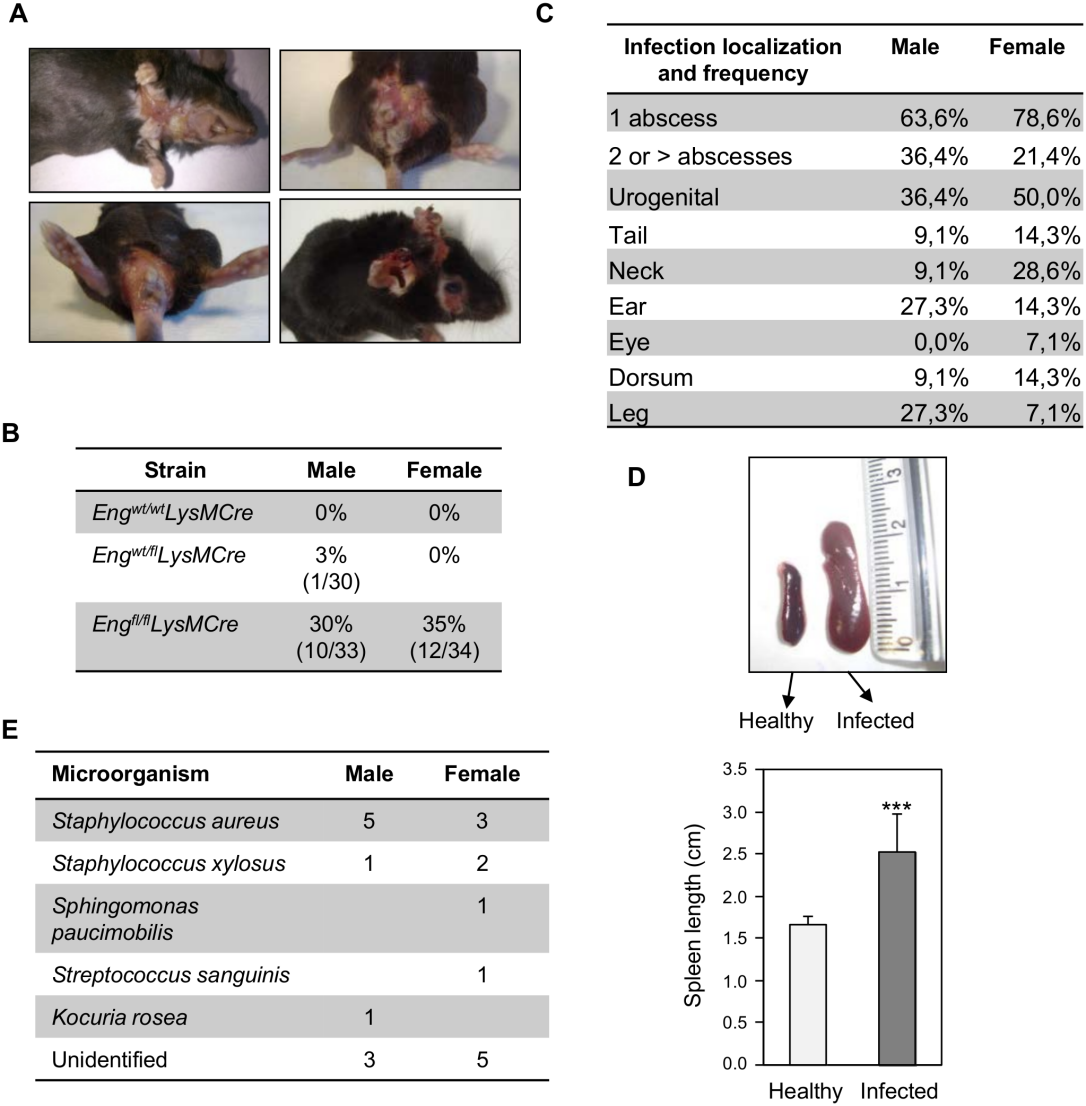
Endoglin deletion in MΦ leads to increased susceptibility to develop spontaneous infections. **(A)** Localization of cutaneous infection in *Eng*^*fl/fl*^*LysMCre* mice. Skin abscesses were observed in abdomen, eyes, ears, legs and dorsum of reproductive *Eng*^*fl/fl*^*LysMCre* mice. One male *Eng*^*wt/fl*^*LysMCre* mice developed infection and no infections were seen in control mice. Infections by *S*. *aureus* are characterized by pyogenic infections of the conjunctiva and eye adnexa, skin and adnexa of the genital tract, as is shown in the pictures. **(B)** Frequency of local infections in reproductive mice. Reproductive *Eng*^fl/fl^*LysMCre* mice destined to maintain the strain developed infection by opportunistic bacteria. Cutaneous infections were only detected in *Eng*^fl/fl^*LysMCre* reproductive individuals, and 1 male *Eng*^*wt/fl*^ *LysMCre* while the control mice remained healthy. **(C)** Frequency and localization of spontaneous infections in *Eng*^*fl/fl*^*LysMCre* mice. Normally, mice present one abscess, most frequently localized around the urogenital area. **(D)** Necropsis of infected individuals revealed that splenomegaly was associated with infections. **(E)** Bacteria isolated from infected areas identified by API strips method.

All the strains were kept in the same room, and under the same breeding conditions. Interestingly, we observed local spontaneous infections in a notable percentage of reproductive individuals (32.5% of animals) of the *Eng*^fl/fl^*LysMCre* genotype, and one of 30 *Eng*^*wt/fl*^*LysMCre* males (Fig 5). The percentage of spontaneous infections reflects the incidence only in adult mice that were interbred to maintain the experimental strains. We only detected one single spontaneous infection in an *Eng*^*fl*^*/*^*fl*^*LysMCre* male housed with its littermates. Infections affected both sexes equally, and the most frequent localization was in the abdominal region surrounding the urogenital area. Usually, when one individual developed an infection, as they were housed in the same cage, they transmitted the infection to their breeding partner. Females underwent a normal gestation, but if infected, they usually ate their litter. Also, if the infection in females appeared immediately after set up, they did not become pregnant. Unfortunately, this led to a decrease of breeding efficiency. Necropsis of infected individuals revealed a splenomegaly secondary to infectious processes in all animals with visible infection symptoms (Fig 5D). To characterize the bacterial strains responsible for the infections, samples from infected animals were assessed and analyzed at the microbiological department of Complutense University (Madrid). Several opportunistic bacteria were identified, but infections were mainly due to *Staphylococcus aureus* (Fig 5E). Therefore, mice with MΦ lacking endoglin show increased susceptibility to infection by opportunistic bacteria.

### Differential survival rate of myeloid specific *Eng* knock-out mice (*Eng*^*fl/fl*^*LysMCre*) to septic shock and altered pro-inflammatory cytokine profile

**Fig 6:**
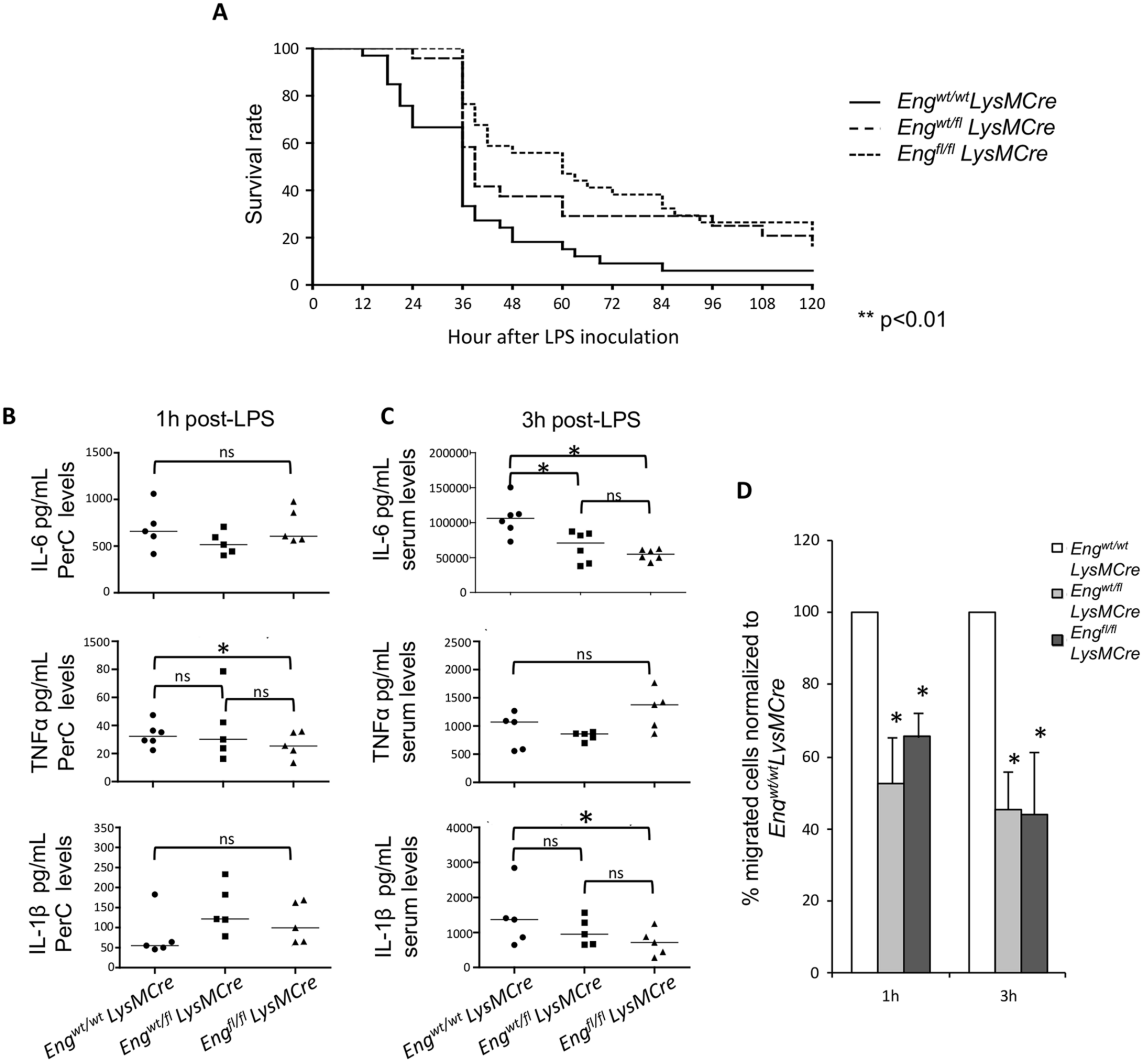
Delayed endotoxin-induced mortality in *Eng*^wt/fl^*LysMCre* and *Eng*^*fl/fl*^*LysMCre* is associated with decreased levels of pro-inflammatory cytokines. **(A)** Survival of *Eng*^*wt/wt*^*LysMCre* (n = 33), *Eng*^*wt/fl*^*LysMCre* (n = 24) and *Eng*^*fl/fl*^*LysMCre* (n = 34) mice after i.p. injection with LPS (40 mg/Kg body weight). The graph represents pooled survival data from three independent experiments. Survival was monitored for 5 days after LPS challenge. **(B)** PerC concentrations of IL-6, TNF-α and IL-1β, determined by ELISA, 60 min after injection of LPS. The median in each group is represented by the horizontal line. **(C)** Serum concentrations of IL-6, TNF-α and IL-1β, determined by ELISA, 3 hours after injection of LPS. The median in each group is represented by the horizontal line. *P<0.05 **(D)** *In vitro* transwell migration of SR.D10.CD4^-^. F1 cells in response to peritoneal exudates from *Eng*^wt/wt^*LysMCre*, *Eng*^wt/fl^*LysMCre* and *Eng*^fl/fl^*LysMCre* mice, at 1 and 3 hours after LPS challenge. Quantification of migrated cells was performed by flow cytometry. Mean ± SEM of 3 independent experiments (N = 3) performed in duplicate, is presented. P<0.05, **P<0.01, ***P<0.001.

As the previous results suggested an immune-compromised phenotype following endoglin deletion in MΦ, we next assessed the primary immune responses in the three genotypes: endoglin KO, heterozygous and controls, to test the role of endoglin in MΦ during the innate immune response. For this purpose a LPS septic shock was induced and survival of animals was monitored for 5 days. Mice with normal MΦ (*Eng*^*wt/wt*^*LysMCre*) were significantly more susceptible to LPS treatment than heterozygous (*Eng*^*wt/fl*^*LysMCre*) and KO (*Eng*^*fl/fl*^*LysMCre*) mice. During the first 36h following the LPS injection, animals lacking endoglin in MΦ showed a delayed endotoxin-induced mortality and a higher survival at the 120 hour endpoint (Fig 6A). Animals alive at the endpoint had completely recovered from the septic shock, with healthy and normal appearance and appetite. At this endpoint, no pain signals were observed.

Since production of pro-inflammatory cytokines is rapidly activated following LPS injection, levels of TNF-α, IL-1β and IL-6 in PerC and in blood serum, were analyzed at early times 1 & 3 hours post-injection, to compare responses between the three genotypes. The absence of endoglin did not affect TNF-α serum concentrations (Fig 6B), but these were significantly lower in PerC 1h post-LPS injection in *Eng*^*wt/fl*^*LysMCre* and *Eng*^*fl/fl*^*LysMCre* mice than in control animals (Fig 6B). On the other hand, the increase of IL-1β and IL-6 serum levels, 3 hours after LPS injection, was significantly lower in *Eng*^*wt/fl*^*LysMCre* and *Eng*^*fl/fl*^*LysMCre* mice compared to controls (Fig 6C).

To functionally support the differences in pro-inflammatory cytokines found in PerC among the different genotypes, a lymphocytic cell line, SR.D10-CD4^-^F1 was used in *in vitro* migration assays to measure the migratory response to peritoneal exudates from the different genotypes. As can be seen in Fig 6D, peritoneal exudates from mice with endoglin deficiency were significantly less effective in recruiting lymphocytes than exudates from control mice.

### Impaired in vivo leukocyte transmigration in *Eng*^*fl/fl*^*LysMCre* mice

**Fig 7:**
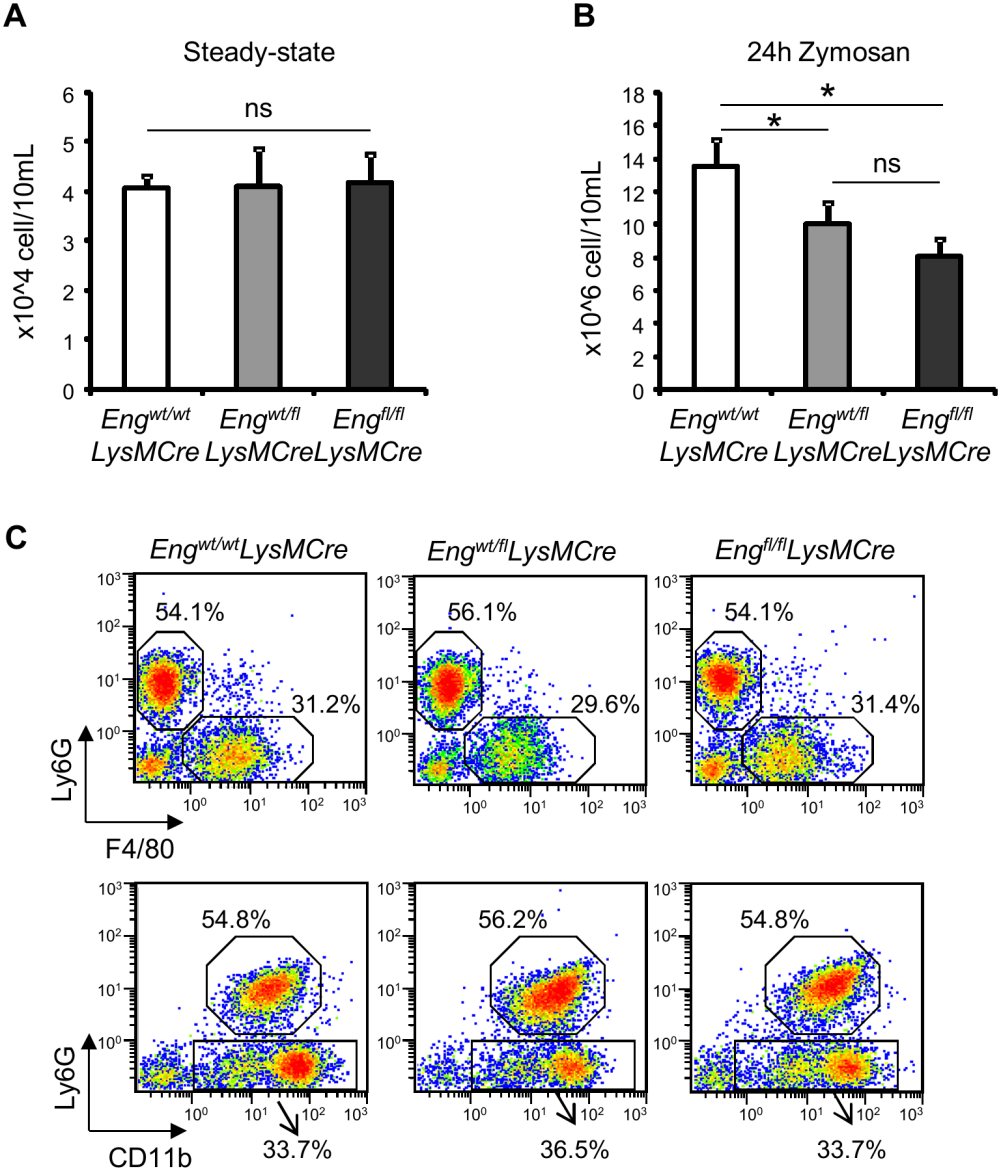
(A) Total number of cells in the PerC from *Eng*^wt/wt^*LysMCre* (n = 9), *Eng*^wt/fl^*LysMCre* (n = 7) and *Eng*^fl/fl^*LysMCre* (n = 11) in steady-state conditions. **(B)** Recruitment to the PerC of inflammatory cells during zymosan-induced peritonitis in *Eng*^wt/wt^*LysMCre*, *Eng*^wt/fl^*LysMCre* and *Eng*^fl/fl^*LysMCre* mice. Animals were i.p. injected with zymosan A (1 mg/500 μL of PBS), humanely euthanized 24 h later, and the peritoneal cells were obtained. Total number of cells recruited 24 h after ZIP in *Eng*^wt/wt^*LysMCre* (n = 9), *Eng*^wt/fl^*LysMCre* (n = 7) and *Eng*^fl/fl^*LysMCre* (n = 11). *p<0.05; ns = non-significant **(C)** Representative FACS profile of F4/80 vs Ly6G, Ly6G vs CD11b staining of total isolated peritoneal cells obtained 24 h after the zymosan challenge. Dot-plots shown in this figure are gated on total live PerC cells. Data are representative of 3 independent experiments.

Because cytokines are chemoattractants that direct leukocytes to sites of inflammation, we next investigated if endoglin expression in MΦ played a role in leukocyte transmigration, in an *in vivo* model of acute inflammation. To this end, peritonitis was induced by injecting Zymosan A in PerC (ZIP). The number of resident PerC cells in quiescence is unaffected by the presence or absence of endoglin expression in MΦ (Fig 7A), and the total number of leukocytes and subpopulations in peripheral blood are similar between the strains (Table 1). However, twenty-four hours after Zymosan challenge, *Eng*^*fl/fl*^*LysMCre* mice show a significantly lower influx of leukocytes to PerC compared to control mice *Eng*^*wt/wt*^*LysMCre* (Fig 7B). The cell influx is mainly represented by blood granulocytes (CD11b^pos^ Ly6G^pos^) and blood Mo differentiated to MΦ (CD11b^pos^ F4/80^low^) (Fig 7C).

**Table 1:** Hematologic analysis of *Eng*^*wt/wt*^*LysMCre* and *Eng*^*fl/fl*^*LysMCre* mice. Numbers shown are means ± SD. Peripheral blood differentials are shown as absolute counts.

Parameter	*Eng*^*wt/wt*^*LysMCre* (n = 5)	*Eng*^*fl/fl*^*LysMCre* (n = 7)	Normal range
White blood cell count (x10^9^/L)	5.67 ± 0.20	5.37 ± 0.13	[6–15]
Lymphocytes, (x10^9^/L)	4.50 ± 0.17	4.08 ± 0.12	[3.4–7.44]
Monocytes, (x10^9^/L)	0.35 ± 0.03	0.34 ± 0.04	[0.0–0.6]
Granulocytes (x10^9^/L)	0.82 ± 0.09	0.78 ± 0.03	[0.5–3.8]
RBC count, (x10^12^/L)	9.16 ± 0.10	9.36 ± 0.02	[7–12]
Haemoglobin, g/L	142.40 ± 2.40	148.95 ± 0.29	[122–162]
Hematocrit %	38.88 ± 0.40	40.86 ± 0.09	[35–45]
Platelets (x10^9^/l)	408.76 ± 11.55	460.34 ± 4.04	[200–450]

### Phagocytosis is impaired in Eng KO macrophages

**Fig 8:**
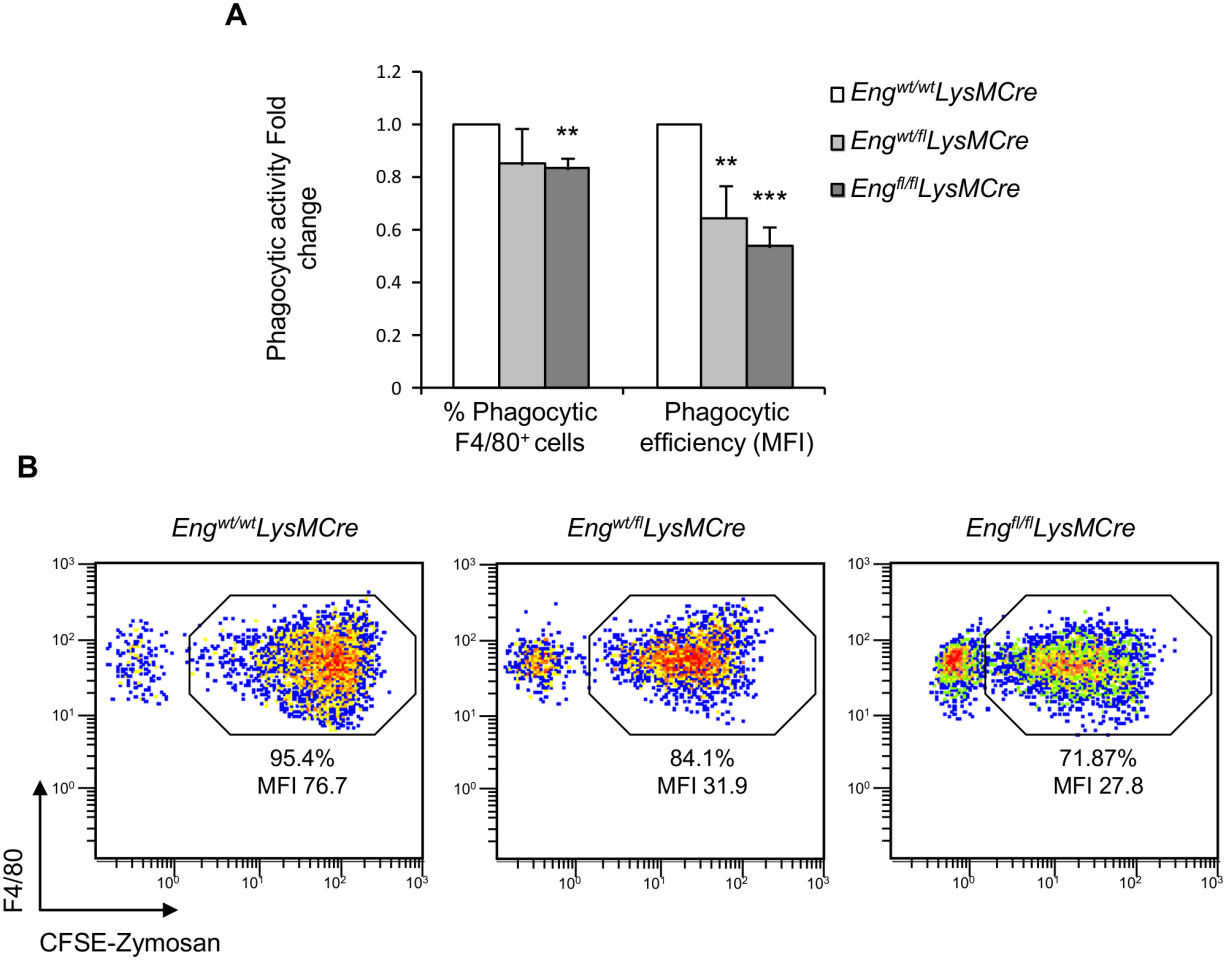
Endoglin deletion in MΦ impairs phagocytosis. **(A)** *In vivo* phagocytic activity of PerC MΦ. Mice were i.p. injected with fluorescent Zymosan particles (50 μg/500μL PBS 1x) for 90 min. The phagocytic activity was evaluated in F4/80^pos^ cells. Phagocytic activity, represented by the percentage of MΦ (F4/80^pos^) that have incorporated Zymosan particles (CFSE^pos^) is decreased in *Eng*^fl/fl^*LysMCre*, as well as the efficiency of phagocytosis defined as the number of particles incorporated per cell and represented by CFSE MFI in F4/80 positive cells. Data were normalized to *Eng*^*wt/wt*^*LysMCre* mice and presented as the mean ± SEM. Results are pooled from 3 independent experiments with each experiment performed in duplicate; data was analyzed versus respective controls using a Student’s *t test* (N = 3). **P<0.01 or ***P<0.001. **(B)** Representative flow cytometry profile of F4/80 staining vs CFSE fluorescence. Phagocytic activity was measured in F4/80^pos^ cells. Gated area shows F4/80^pos^ cells that have incorporated Zymosan-CFSE particles. Percentage of phagocytic cells and the MFI are shown.

Phagocytosis can be measured by the incorporation of fluorescently labeled Zymosan A particles by resident LPM. When endoglin was reduced or absent, MΦ exhibit deficient phagocytosis of Zymosan particles (Fig 8). Phagocytic activity (represented by the percentage of MΦ that have incorporated fluorescent particles; positive for CFSE signal) and phagocytic efficiency (represented by the CFSE Mean Fluorescence Intensity (MFI)) in F4/80^pos^ cells were both decreased in MΦ from *Eng*^*fl/fl*^*LysMCre* compared to control mice (*Eng*^*wt/wt*^*LysMCre*). In heterozygous mice, the phagocytic efficiency is also clearly decreased compared to control mice.

### Endoglin alters the expression of TGF-β target genes in MΦ

**Fig 9:**
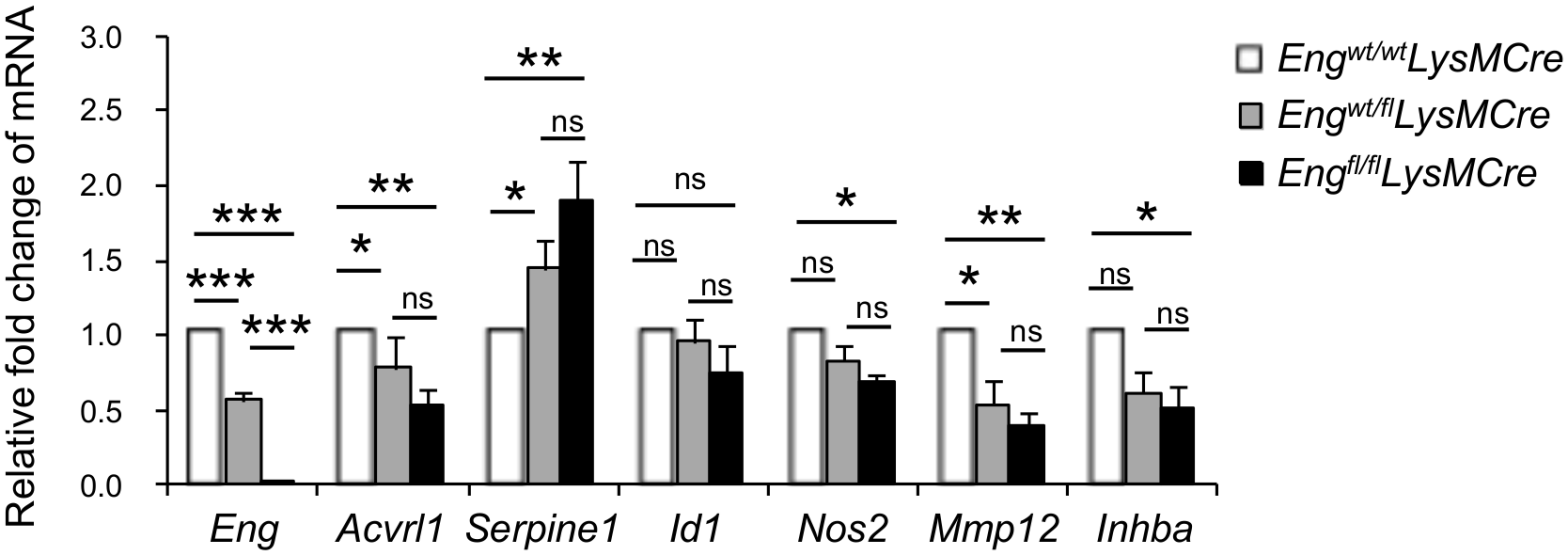
Endoglin deficiency alters the pattern of expression of TGF-β target genes. Real-time PCR for *Eng*, *Acvr1*, *Serpine1*, *Id1*, *Nos2*, *Mmp12* and *Inhba* in cultured peritoneal MΦ. Relative mRNA levels are normalized to *18S* as endogenous controls. Expression in *Eng*^*wt/wt*^*LysMCre* is considered the basal level. Data are shown as mean ± SEM from 5 independent experiments (N = 5). * P<0.05, ** P<0.01 *** P<0.001.

As endoglin is a TGF-β1 co-receptor, we evaluated the expression of selected downstream genes: *Acvrl1*, *Serpine1*, *Id1*, *Nos2*, *Mmp12* and *Inhba* in *in vitro* cultures of control, heterozygous and KO PerC MΦ (Fig 9). Macrophages were selected by adherence to plastic flasks and cultured in DMEM supplemented with 10% FCS for 24 hours. Gene expression was also checked in untreated cultured macrophages. Endoglin deficiency in MΦ led to a reduced expression of *Acvrl1* and *Mmp12* genes. We also observed reduced expression of *Nos2* and *Inhba* in endoglin-deficient MΦ. Of note, there was a trend suggesting intermediate loss of target gene expression in MΦs that were heterozygous for endoglin expression with levels of *Nos2* and *Inhba* significantly reduced compared with controls. *Serpine1* expression in MΦs from *Eng*^*fl/fl*^*LysMCre* mice was significantly increased compared to those of *Eng*^*wt/fl*^*LysMCre* or *Eng*^*wt/wt*^*LysMCre*. No statistically significant changes in *Id1* expression were detected.

## Discussion

Endoglin is expressed during the *in vitro* differentiation of human Mo [18, 19, 25], but its expression in murine MΦ has remained elusive and controversial. Some authors could not detect endoglin by immunohistochemistry in murine atherosclerotic plaques [34] and had considered that murine MΦ do not express endoglin. However, more recently, endoglin transcripts in murine MΦ were reported by semi-quantitative RT-PCR [35]. The results reported in the present manuscript, show the expression of endoglin in murine MΦ in three different contexts: (i) during *in vitro* differentiation of Mo, (ii) during *in vivo* transition of peripheral blood Mo to Mɸ following an inflammatory process, and (iii) on tissue-resident Mɸ isolated from liver and peritoneal cavity of untreated C57BL/6 mice. Taken together this shows that endoglin is expressed in murine MΦ and that endoglin is also a marker of Mo differentiation towards MΦ, since endoglin is not detected on circulating Mo (Fig 1A and 1B). However, the endoglin levels that we found on differentiated blood Mo to MΦ in mouse are lower (22% of murine Mo were endogline positive) compared to those reported for cultured human Mo, where 94% of differentiated MΦ are positive for endoglin expression after 21h in culture [25].

Endoglin is expressed in terminally differentiated M1/M2 macrophages. In this context, Aristorena and colleagues [35] recently demonstrated that overexpression of S-endoglin in U937 cells (a human promonocytic cell line) impairs differentiation to the pro-inflammatory M1 phenotype. S-endoglin counteracts the signaling prompted by L-endoglin via ALK1/Smad1/5 [36]. These data suggest that L-endoglin would be necessary for the complete differentiation, at least to M1 phenotype. On the other hand, we have seen that both M2 and M1 macrophages express endoglin, suggesting that endoglin expression is associated with a complete M2 or M1 phenotype.

The expression of endoglin during Mo *in vivo* differentiation is compatible with the involvement of endoglin in cellular trafficking to the target tissues. Previous studies described that endoglin on endothelial cells is involved in adhesion to the extracellular matrix [37], and in promoting leukocyte adhesion to vascular endothelium [38]. Both processes are involved in inflammation and leukocyte extravasation, however, the role of MΦ endoglin in innate immunity and inflammation is not yet well established. Constitutive overexpression of endoglin in U937 cells showed the deregulation of hundreds of genes compared to the parental line. These genes are involved in cellular movement, cell adhesion and transmigration [35, 39]. In the present work, we have followed *in vivo* endoglin expression in SPM and LPM (the two macrophage subsets present in PerC) during leukocyte recruitment induced by a ZIP process. Subsequently to Zymosan challenge, granulocytes are the predominant myeloid population in PerC, and they did not express endoglin. Following Zymosan injection, MΦ seem to disappear from PerC. The “macrophage disappearance reaction” is attributed to their migration to the omentum [40]. Following the granulocytes influx after Zymosan challenge, recruited peripheral blood Mo differentiate to SPM being the predominant MΦ population in PerC. A discrete LPM population is detected 24 hours after ZIP and seems to be derived from SPM. This new LPM population starts to become positive for endoglin expression 3 days after ZIP, as do SPM cells. Thus, we suggest that SPM contribute to the replenishment of LPM after ZIP, similar to other situations where Mo-derived MΦ make substantial contribution to the population of resident MΦ [41]. Moreover, the results shown in this work indicate that endoglin is a marker of differentiated MΦ, suggesting that endoglin could affect early inflammatory events orchestrated by resident MΦ, not from Mo during the process of transmigration, although a transient endoglin up-regulation during this event cannot be ruled out. Nonetheless, caution is required when comparing *in vitro* and *in vivo* data, and our results indicate that up-regulation of endoglin during Mo to MΦ differentiation *in vivo* is much slower than Mo differentiation *in vitro*.

To elucidate the role of endoglin in differentiated MΦ a mouse strain lacking endoglin in MΦ was generated using the *LysMCre* model. The value of myeloid-specific promoters in transgenic mice has been discussed since knock-out of MΦ markers such as CD11b, CD11c and F4/80 has no impact on MΦ numbers and remarkably, little impact on MΦ function [30]. The *LysMCre* models do not really allow a distinction among myeloid cell types since Cre is expressed in Mo, MΦ and granulocytes. Cre-mediated excision is effective in the majority of MΦ and granulocytes but considerably lower in CD11c^+^ DCs [30]. In our *LysMCre* model, we have not seen an alteration on resident PerC MΦ numbers but we have observed effective action of Cre recombinase on endoglin floxed gene leading to reduced phagocytosis, one of the main functions of resident MΦ.

Endoglin deletion in MΦ predisposes animals to develop infections by opportunistic bacteria, where *S*. *aureus* is the most predominant pathogen identified. In immunocompetent animals, *S*. *aureus* colonization of the skin, intestinal tract, or nasopharynx is generally asymptomatic while in immunocompromised or immunodeficient animals, may cause pyogenic (abscess) infections. In our model, mice lacking endoglin on MΦ (*Eng*^*fl/fl*^*LysMCre*) were susceptible to develop infections by opportunistic bacteria after being set up in breeding pairs. We postulate that minor wounds due to the physical interactions between mice prior to and during mating were responsible for the development of infections in *Eng*^*fl/fl*^*LysMCre* mice. The low incidence of spontaneous infections in *Eng*^*wt/fl*^*LysMCre* mice may be due to a threshold effect where endoglin has to fall below a critical level in MΦ in order to show the infectious phenotype. In this regard, mouse models of HHT suggest that AVMs in HHT patients occur following loss of heterozygosity [26]. All mice were maintained in the same room and the same conditions, but environmental factors could be relevant in determining the type and frequency of infections.

Mice lacking endoglin in MΦ show other characteristics compatible with an immunocompromised phenotype. The lack of endoglin in MΦ impairs phagocytosis, and this may be affecting the initiation of the innate immune response. Upon bacteria recognition and phagocytosis, MΦ orchestrate coordinated inflammatory responses involving recruitment of neutrophils and other inflammatory cells [42]. *Eng*^*fl/fl*^*LysMCre* mice, and to a lesser extend *Eng*^*wt/fl*^*LysMCre* mice, showed a deficient recruitment of inflammatory cells to sites of infection. These differences may be influenced by the absence or deficiency of endoglin expression in resident PerC MΦ. Furthermore, animals with reduced or absent endoglin expression in MΦ showed an extended survival in the first hours following induction of septic shock. and a reduced production of inflammatory cytokines. Altogether, these data suggest that endoglin expression in resident MΦ is relevant for initiation of the innate immune response.

While endoglin plays a pivotal role modulating TGF-β signaling pathways on endothelial cells, its role on TGF-β signaling in MΦ is not well known. Stable transfectants of two different alternatively spliced isoforms of endoglin in the human promonocytic cell line U937 showed that endoglin isoforms counteracts TGF-β1 inhibition of proliferation and migration [3] and displayed a differential gene expression pattern, mainly affecting biological function related to cell movement and the expression of *INHBA*, a TGF-β family member [39]. In the immune system, TGF-β initially plays a pro-inflammatory role, acting as a chemoattractant for Mo, and triggering the production of inflammatory mediators. However, TGF-β later functions mainly as an inhibitory molecule, when Mo differentiate into MΦ. This TGF-β inhibitory function contributes to resolve inflammation and prevents the development of immunopathologies [17]. Due to their involvement in the TGF-β signaling pathways, the expression of two relevant target genes of TGF-β; *Serpine1* (Pai-1 gene) and *Id1*, controlled by TGF-β/ALK5/Smad2/3 and by TGF-β/ALK1/Smad1/5/8 pathways in endothelium, respectively, were analyzed. While, *Id1* expression seems to be independent of endoglin, *Serpine1* is highly expressed on MΦ lacking endoglin compared to MΦ from control mice. This overexpression is in agreement with previous reports where endoglin and PAI-1 levels are inversely correlated [43]. Since Smad3 is a critical mediator of TGF-β inhibition of MΦ activation [44], these results suggest that the absence of endoglin could act by increasing TGF-β signaling via ALK5/Smad2/3 on MΦ. Moreover, haploinsufficiency or absence of endoglin in MΦ leads to the repression of *Acvrl1* expression. These data agree with previous reports where endothelial cells from HHT1 patients also show a downregulation of *ACVRL1* expression [45].

Our observations of decreased levels of *Nos2*, *Mmp12* and *Inhba* transcripts in MΦ with reduced endoglin expression, would further strengthen the idea that endoglin counteracts in MΦ the known inhibitory effects of TGF-β1 signaling through the ALK5/Smad2/3 pathway. Furthermore, Nos2, MMP12 and Activin A are all markers of MΦ activation [44, 46]. Nos2 expression helps to control bacterial infections such as *S*. *aureus* [47] and MMP12 plays a role in early killing of *S*. *aureus* in the phagolysosome of MΦ [48]. Thus, the decreased expression of *Nos2* and *Mmp12* in *Eng*^*fl/fl*^*LysMCre* mice would impair bacterial clearance by MΦ.

The decreased capacity for MΦ activation seen in *Eng*^*fl/fl*^*LysMCre* mice is compatible with a weaker primary immune response. The affected immune functions in *Eng*^*fl/fl*^*LysMCre* mice suggest a possible explanation for certain infectious events seen in HHT patients [21, 22] such as rare infections, osteomyelitis, sepsis and extracerebral abscesses, among others. In fact, it has been reported that HHT patients display abnormalities of phagocytosis and oxidative burst exerted by neutrophils and Mo [49], although another study did not find this impairment [22]. An explanation for this discrepancy is that patients with HHT exhibit a great diversity of clinical manifestations due to an incomplete penetrance of the disease and the influence of environmental factors. In addition, the cellular types analyzed are not the most appropriate since neutrophils do not express endoglin and in blood Mo, it is almost undetectable [18, 20]. Future studies on a larger patient cohort and focusing on differentiated MΦ, would be more suitable to determine the effects of endoglin mutations on the innate immune response in HHT1 patients. The inclusion in the international guidelines of immunological assessment during management of HHT patients would be useful to prevent serious infectious outcomes. In this context, preventive protocols for vaccination [50] and review of antibiotic prophylaxis for hospitalized HHT patients should improve their clinical management and outcomes.

## Materials and Methods

### Experimental animals

Specified pathogen-free C57BL/6 male 10-12-week-old mice were used in the experiments. Mice were housed under specific pathogen-free conditions at the department of Animal Resources facilities in the Centro de Investigaciones Biológicas (CSIC). *LysMCre* mice were provided by Dr. Mercedes Ricote (Fundación Centro Nacional de Investigaciones Cardiovasculares, Madrid, Spain). Endoglin floxed mice (*Eng*^*fl/fl*^) were generated as described [51] and were crossed with *LysMCre* individuals to generate mice with specific *Eng* gene deletion in the myeloid lineage. The first heterozygous offspring containing the loxP-targeted *Eng* gene (*Eng*^*wt/fl*^) and the Cre transgene (*LysMCre*) were backcrossed for 10 generations selecting heterozygous individuals (*Eng*^*wt/fl*^*LysMCre*) to achieve homogeneity. The offspring *Eng*^*wt*^*/*^*wt*^*LysMCre* resulting from the 10th generation of backcrosses between *Eng*^*wt*^*/*^*fl*^*LysMCre* mice were then interbred to increase the number of control mice (*Eng*^*wt/wt*^*LysMCre*). *Eng*^*wt/wt*^*LysMCre* were crossed with *Eng*^*fl/fl*^*LysMCre* mice to obtain the heterozygous experimental animals (*Eng*^*wt/fl*^*LysMCre*), and *Eng*^*fl/fl*^*LysMCre* individuals were interbred to maintain the strain *Eng*^*fl/fl*^*LysMCre*. Mice were genotyped by PCR using the primers X 5’-CCACGCCTTTGACCTTGC 3’, Y 5’-GGTCAGCCAGTCTAGCCAAG 3’, Z 5’-GTGGTTGCCATTCAAGTGTG 3’ as described [51] and primers Cre Fw 5’-AGGTGTAGAGAAGGCACTTAGC 3’ and Cre Rv 5’-CTAATCGCCATCTTCCAGCAGG 3’. DNA was extracted from tails using the REDExtract-N-Amp Tissue PCR Kit (Sigma #XNAT). From PerC MΦ and different tissues, DNA was obtained using QIAamp DNA Mini Kit (QIAGEN #51304).

### Isolation of cells

For isolation of peripheral blood cells, blood samples were obtained by cardiac puncture using heparin as anticoagulant. Blood samples were treated twice with red blood cell lysis buffer (1g/L KHCO_3_, 8.3g/L NH_4_Cl, 0.019% EDTA) for 2 min at RT. Samples from a total of 10 mice were pooled. Mo were isolated by incubating the total blood leukocyte fraction at 37°C and, 5% CO_2_, in autologous plasma-coated plastic flasks. Non-adherent cells were removed by extensive washing with a pre-warmed Hanks’ solution. Adherent cells were trypsinized at selected times. For isolation of Kupffer cells, liver was removed from the PerC and rinsed in Krebs-Ringer-Buffer (KRB-1000; Zen-Bio Inc., NC, USA). To obtain a single-cell suspension from mouse liver, the gentle MACS Dissociator (#130-095-937; Miltenyi Biotec GmbH, Bergisch Gladbach, Germany) was used following the manufacturer’s guide, using collagenase IV treatment (C5138; Sigma-Aldrich, Saint Louis, MO, USA). For collection of PerC cells, 10 mL of PBS were injected in the PerC. After an abdominal soft massage, between 8.5 to 9.5 mL were recovered. The suspension obtained from peritoneal lavage was centrifuged at 1,200 rpm for 5 min to recover cells. Blood contaminated samples were discarded.

### Cell culture and treatments

Bone Marrow-Derived Macrophages (BMDM) were obtained by flushing mouse femurs with ice-cold PBS. From 5 to 8x10^6^ cells, were cultured in DMEM supplemented with 10% heat-inactivated FCS and 50μM β-mercaptoethanol, containing human macrophage CSF (M-CSF) (25 ng/mL) or murine GM-CSF (1000U/mL; ImmunoTools GmbH), respectively, for 7 days to obtain 95%-pure CD11b^pos^ BMDMs. Cytokines were added every two days. After this, media were discarded and cells rinsed twice with saline solution. Peritoneal MΦ were harvested from *Eng*^*wt/wt*^*LysMCre*, *Eng*^*wt/fl*^*LysMCre* and *Eng*^*fl/fl*^*LysMCre* mice as described above. After 1 hour of incubation at 37°C, non-adherent cells were discarded by extensive washes with warm Hanks’ solution and then incubation was continued for 24h at 37°C. SR.D10-CD4^neg^F1 was a CD4^neg^ mutant cell line cloned from the mouse CD4^pos^ TH2 cell line D10.G4.1 [52]. It was grown in Click medium with 10% FCS, 9% (v/v) β-mercaptoetanol, 5 U/mL IL-2; 10 U/mL IL-4, and 25 pg/mL IL-1α.

### Hematological analysis

Peripheral blood was obtained by puncturing the cava vein. Blood was drawn into EDTA-coated tubes (Monlab SL, Spain). Complete blood profiles and hemoglobin levels were obtained using an Abacus Junior Vet (Diatron) hematology analyzer. Values are shown as absolute counts and referenced against the normal range established for mice.

### Zymosan induced peritonitis (ZIP)

Ten week-old mice were i.p. injected with 1 mg of Zymosan A from *Saccharomyces cerevisiae* (Z4250; Sigma-Aldrich) in 0.5 mL of sterile PBS. Isolation of PerC cells was performed as described above. For *in vivo* evaluation of endoglin expression on MΦ surface, samples were collected at 12h, 24h, 36h, 3, 7 and 14 days after injection of Zymosan A, and stained for flow cytometry analysis. Samples of unstimulated mice were used as time 0. For other experiments, animals were similarly i.p. injected with 1 mg of Zymosan A in 0.5 mL of sterile PBS. Twenty-four hours later, PerC exudates were recovered to evaluate the leukocyte recruitment to the PerC after ZIP. The number of total leukocytes in PerC was evaluated in a CASY Cell Counter. Events were considered leukocytes above a threshold of 5.7 μm diameter. Percentage of different leukocyte subpopulations in PerC was evaluated by flow cytometry.

### Flow cytometry

BMDMs and cell suspensions were blocked with PBS containing 5% of rabbit serum for 20min at 4°C, followed by incubation with an Ab against endoglin (eBioscience, 14–1051) or rat anti-mouse isotype control (eBioscience, 14–4321) for 1h at 4°C. Thereafter, cells were washed twice with 1% BSA in PBS and incubated with a FITC-conjugated F(ab’)_2_ rabbit anti-rat IgG (Invitrogen A11006) for 20 min at 4°C. After endoglin staining, cell suspensions were washed twice and incubated at 4°C during 20 min with the following monoclonal antibodies: PE anti-mouse F4/80 (Biolegend; 122616), PE anti-CD11b (Immunostep; M11BPE), APC anti-mouse Ly6G (Immunostep; MLY6GA), anti-mouse CD19 FITC (Immunostep; M19F), anti-mouse CD3e FITC (Immunostep; 220911). For isotype controls, antibodies were: PE rat IgG2b, κ (Biolegend; 400608), rat IgG2a APC (Immunostep; 220812/RIGG2A) and Alexa 488 rat IgG2a, κ (Biolegend; 400525). Unbound antibodies were removed by washing twice with PBS containing 1% of BSA. Flow cytometry analyses were performed with a Beckman Coulter FC500 cytometer. Cells were first selected on the basis of their FS vs SS properties. Dead cells were localized by propidium iodide (Sigma #81845) exclusion to set the gating area of interest. A minimum of 5,000 stained cells per sample was analyzed. Upon gating, levels of endoglin were analyzed on the CD11b^pos^ Ly6G^neg^ CD3^neg^ population of adherent cells for *in vitro* assay and on the F4/80^pos^ Ly6G^neg^ CD19^neg^ of cell suspensions from liver and PerC. Flow cytometry experiments were carried out by fitting isotype controls to the first decade on log histograms, setting upper limit at 10°. A residual percentage of positive cells lower than 5% above this limit was considered as a negative control. Endoglin expression is represented by the percentage of positive cells, and cells are considered positive for endoglin expression when the population is over 5%.

### Immunohistochemistry

Immediately after sacrifice, mice were perfused with freshly prepared 1% paraformaldehyde (PFA). Liver was excised and fixed in 1% PFA for 12h at 4°C, and 15% and 30% sucrose solution, until the specimens were decanted, and frozen in OCT. Cryosections were incubated with an Ab against endoglin (eBioscience, 14–1051) or rat anti-mouse isotype control (eBioscience, 14–4321, overnight at 4°C. Endoglin was detected following 1 hour incubation with Alexa 488 anti-rat (Molecular Probes #A-11006). All the incubations were done in the presence of 5% goat serum in PBS. Staining was visualized by laser confocal scanning microscopy (TCS-SP2-AOBS; Leica).

### sEng serum levels

For sEng measurements, serum from 10–12 week old mice isolated from cava vein was used. Blood samples were collected and centrifuged at 2,000xg for 20 minutes to collect serum from whole blood. Serum was collected and kept at -20°C until analysis. The levels of sEng were determined using the Mouse Endoglin/CD105 Quantikine ELISA sandwich kit (R&D Systems #MNDG00), following the manufacturer’s guide.

### Microbiological analysis

Individuals with spontaneous infections were sacrificed. Infected areas were cleaned under sterile conditions with sterile PBS, excised and transferred to sterile eppendorf tubes and kept at 4°C. Samples were sent to the Microbiology department of Clinical Veterinary Hospital (Complutense University, Madrid). Isolation of microorganisms was carried out by selective media and identification was achieved by API strips. Spleens were excised from the PerC and rinsed in PBS. Spleen length was measured in all animals suspected of infection (n = 22) and in 10 healthy *Eng*^fl/fl^*LysMCre* mice.

### Septic shock to LPS

Twelve-week-old mice of each genotype were i.p. injected with 40 mg/kg LPS (*E*. *coli* 0111;B4; Sigma). The survival rate was followed for 5 days in *Eng*^*wt/wt*^*LysMCre* (n = 33), *Eng*^*wt/fl*^*LysMCre* (n = 24) and *Eng*^*fl/fl*^*LysMCre* (n = 34) mice. TNF-α, IL-1β and IL-6 levels were analyzed 1 hour after ZIP in PerC samples, and 3 hours post-ZIP in serum samples. Blood samples were obtained by puncture of posterior cava vein, and centrifuged at 2,000g for 20 min at 4°C to obtain serum samples. PerC exudates were obtained by i.p. injection of 10 mL of sterile PBS. Between 8.5–9.5 mL were recovered. TNF-α, IL-1β and IL-6 levels were quantified with ELISA kits (Quantikine R&D Systems).

### Chemotaxis assay

1.5 x 10^5^ SR.D10-CD4^neg^ lymphocytes in a final volume of 100μL of serum free DMEM were placed on the upper compartment of individual Transwell (Costar) chamber wells with pores of 5 μm diameter. Cells were allowed to migrate for 3 hours towards PerC exudates from *Eng*^*wt/wt*^*LysMCre*, *Eng*^*wt/fl*^*LysMCre* and *Eng*^*fl/fl*^*LysMCre* mice. Cells migrating to the lower compartment were counted by flow cytometry. The percentage of migration was normalized to exudates from control mice (*Eng*^*wt/wt*^*LysMCre*).

### Phagocytosis assays

Zymosan particles at a final concentration of 1mg/mL in PBS were CFSE labelled (45 μM) for 15 min at RT, washed three times with PBS and sonicated in RPMI DMEM during 15 min before assay. Mice were i.p. injected with 50 μg of CFSE-stained zymosan particles in 500μL of sterile PBS. After 90 min, mice were anesthetized to isolate PerC exudates. Cellular suspension was processed for F4/80 flow cytometry detection. Phagocytic activity was calculated as the percentage of PerC MΦ that incorporated CFSE-Zymosan particles (F4/80^pos^CFSE^pos^). Phagocytic efficiency represents the Mean Fluorescence Intensity (MFI) of CFSE in F4/80^pos^ cells.

### RNA isolation and Q-PCR

Total cellular RNA was extracted using the NucleoSpin RNA II (Macherey-Nagel, Düren, Germany). Six hundred nanogram of total RNA was reverse transcribed in a final volume of 20 μL with the Kit First Strand cDNA Synthesis (Roche, Mannheim, Germany), using random primers. One μL of fresh cDNA was subjected to real time PCR (in triplicate) using Sybr-green master mix from Bio-Rad. Transcripts of *Eng*, *Acvrl1*, *Serpine1*, *Id1*, *Nos2*, *Mmp12*, *Inhba* and *18s* (as endogenous control), were amplified using specific primers with the following sequences:

*Eng*    Fw: 5’ CGATAGCAGCACTGGATGAC 3’

     Rv: 5’ AGAATGGTGCCTTTGGGTCT 3’

*Acvrl1*   Fw: 5’ TGACCTCAAGAGTCGCAATG 3’

     Rv: 5’ CTCGGGTGCCATGTATCTTT 3’

*Serpine1*   Fw: 5’ GTCTTTCCGACCAAGAGCAG 3’

     Rv: 5’ GACAAAGGCTGTGGAGGAAG 3’

*Id1*      Fw: 5’ GCGAGATCAGTGCCTTGG 3’

     Rv; 5’ CTCCTGAAGGGCTGGAGTC 3’

*Inhba*     Fw: 5’ ATCATCACCTTTGCCGAGTC 3’

     Rv: 5’ TCACTGCCTTCCTTGGAAAT 3’

*Nos2*      Fw: 5’ TGGCCACCAAGCTGAACT 3’

     Rv: 5’ TTCATGATAACGTTTCTGGCTCT 3’

*Mmp12*     Fw: 5’ CCACTTCGCCAAAAGGTTTA 3’

     Rv: 5’ GGGGTAAGCAGGGTCCAT 3’

*18s*     Fw; 5’-CTCAACACGGGAAACCTCAC 3’

     Rv: 5’- CGCTCCACCAACTAAGAACG 3’.

A pool of RNA from 3 individuals was used for each condition. The experiment was repeated five times.

### Statistics

Data were analyzed using SPSS version 11.0.0 (SPSS Inc., Chicago, IL). Reported values are expressed as median and SEM unless otherwise described. Comparisons between control (*Eng*^*wt/wt*^*LysMCre*) and experimental mice (*Eng*^*wt/fl*^*LysMCre* and *Eng*^*fl/fl*^*LysMCre*) were made using ANOVA. Bonferroni’s post hoc multiple comparisons testing was performed to detect significant differences. All comparisons were two-tailed. Survival rates were represented as a Kaplan–Meier curve, and the results were analyzed with a log-rank (Mantel–Cox) t test. Statistical significance is displayed as *(P<0.05), **(P<0.01) or ***(P<0.001).

### Study approval

Mice were maintained under specific pathogen-free conditions at department of Animal Resources facilities in the Centro de Investigaciones Biológicas (CSIC). All animals were handled in strict accordance with good animal practice as defined by the national animal welfare bodies (RD 1201/2005 BOE #252). The experimental design and all animal work were approved by our institutional Ethical Committee following the guidelines of EU Directive 2010/63/UE for animal experiments. For the isolation of organs and body fluids, all animals were deeply anesthetized by isoflurane and sacrificed by cervical dislocation.

## Supporting Information

S1 Fig (A) Schematic representation of the strains used and crossed to obtain the endoglin specific KO mice in myeloid lineage. The strain expressing Cre recombinase from the endogenous *Lyz2* locus (LysMCre) was crossed with the strain containing the floxed endoglin gene (*Eng*^*fl/fl*^). Heterozygous mice for *Eng* floxed allele and positive for *Cre recombinase* were identified and crossed to obtain the three genotypes of interest. **(B)** Schematic representation of Cre recombinase action on endoglin floxed gene. Cre-mediated recombination results in deletion of the flanked sequence by LoxP sites in the myeloid cell lineage, including Mo, mature MΦ, and granulocytes. Cre action results in the deletion of exons 5–6 of endoglin gene. **(C)** Identification of mice genotypes by genomic PCR. Genomic PCR was performed with DNA from tails. The floxed endoglin allele (*Eng*^*fl*^) was recognized by genomic PCR rendering a 566 bp product with primers Y and Z, and discriminated from the 411 bp product corresponding to the WT allele (*Eng*^*wt*^) [51]. The endoglin allele showing the exon 5–6 deletion (*Eng*^5-6^) was detected by genomic PCR which gives rise to a 602 bp product using primers X and Y [51]. **(D)** Efficiency of LysMCre-mediated lox P recombination in different tissues and PerC MΦ. PCR analysis of genomic DNA isolated from the indicated tissues of the three genotypes. The predicted amplicon sizes are indicated. The product of the amplification of *Eng*^Δ*5–6*^ is undetectable in samples of *Eng*^*wt/wt*^*LysMCre* mice. PerC = peritoneal cavity.
(TIF)
