# Supplementary figures and images for: Mice Lacking Endoglin in Macrophages Show an Impaired Immune Response
Source: PLoS Genet. 2016 Mar 24;12(3):e1005935. doi: 10.1371/journal.pgen.1005935 (PMC4806930; doi:10.1371/journal.pgen.1005935)

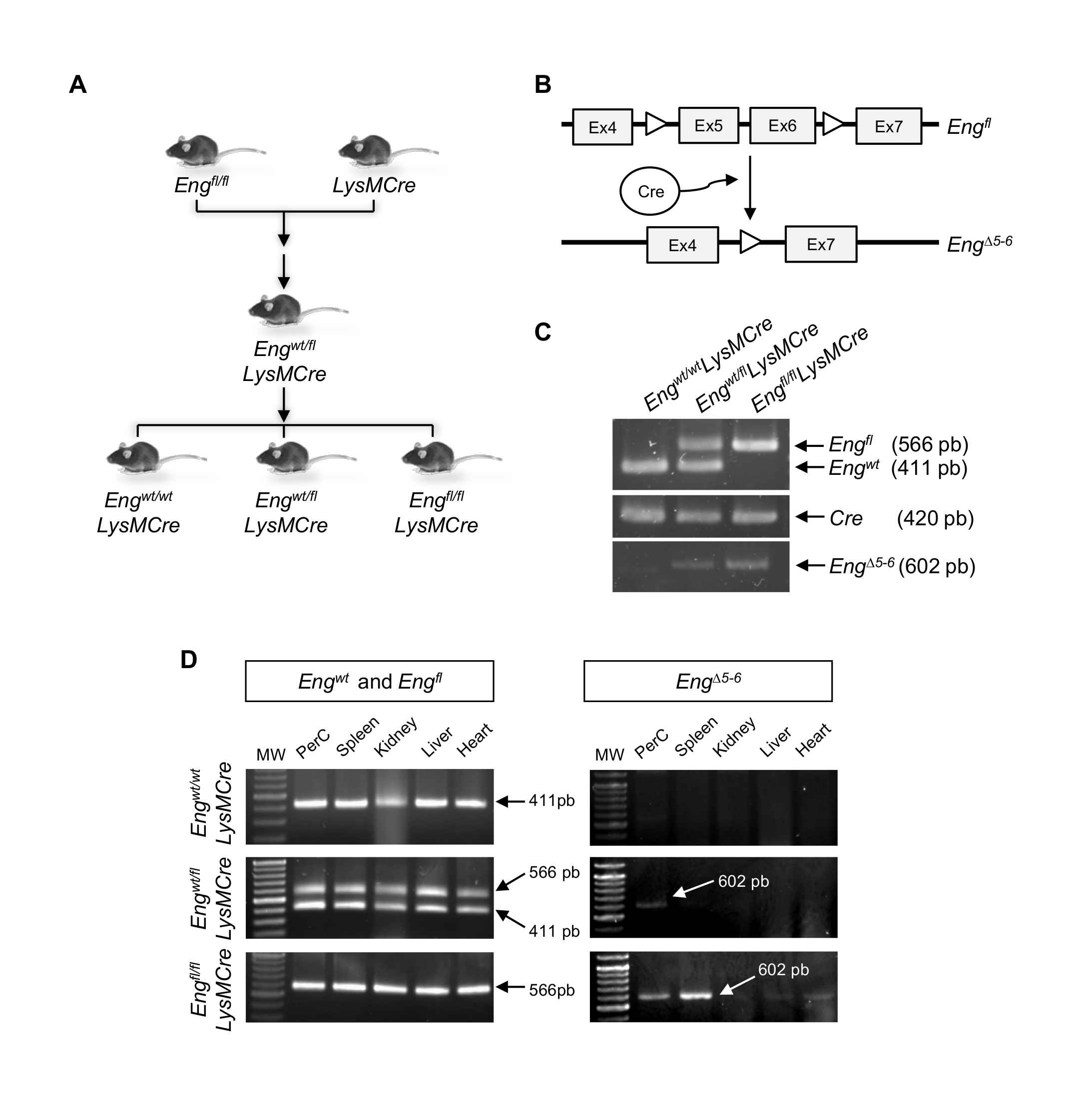

Supplement: S1 Fig — The strain expressing Cre recombinase from the endogenous Lyz2 locus (LysMCre) was crossed with the strain containing the floxed endoglin gene (Engfl/fl). Heterozygous mice for Eng floxed allele and positive for Cre recombinase were identified and crossed to obtain the three genotypes of interest. (B) Schematic representation of Cre recombinase action on endoglin floxed gene. Cre-mediated recombination results in deletion of the flanked sequence by LoxP sites in the myeloid cell lineage, including Mo, mature MΦ, and granulocytes. Cre action results in the deletion of exons 5–6 of endoglin gene. (C) Identification of mice genotypes by genomic PCR. Genomic PCR was performed with DNA from tails. The floxed endoglin allele (Engfl) was recognized by genomic PCR rendering a 566 bp product with primers Y and Z, and discriminated from the 411 bp product corresponding to the WT allele (Engwt) [51]. The endoglin allele showing the exon 5–6 deletion (Eng5-6) was detected by genomic PCR which gives rise to a 602 bp product using primers X and Y [51]. (D) Efficiency of LysMCre-mediated lox P recombination in different tissues and PerC MΦ. PCR analysis of genomic DNA isolated from the indicated tissues of the three genotypes. The predicted amplicon sizes are indicated. The product of the amplification of EngΔ5–6 is undetectable in samples of Engwt/wtLysMCre mice. PerC = peritoneal cavity. (TIF) [file pgen.1005935.s001.tif]
